# Supplementary material for: Impact of Covid-19 on informal employment: A case study of women domestic workers in Khyber Pakhtunkhwa, Pakistan
Source: PLoS One. 2022 Dec 6;17(12):e0278710. doi: 10.1371/journal.pone.0278710 (PMC9725122; doi:10.1371/journal.pone.0278710)
Supplement: S1 Appendix — (DOCX) [file pone.0278710.s001.docx]

**Appendix 1**

**Verbal Informed Consent for Participation (Interview)**

**To be read out by the researcher:** The purpose of the present research study, **“Impact of Covid-19 on Informal Employment: A case study of women domestic workers in Khyber Pakhtunkhwa, Pakistan”** is primarily to investigate that how COVID-19 impacted the life of domestic women workers engaged in informal sector in the province of Khyber Pakhtunkhwa. The study is centered around on how the lockdown disrupted the life of the domestic women workers, how the pandemic affected their livelihoods, what economic hardships they have experienced during the pandemic, what type of social and economic support they received in this unprecedented situation, and what challenges they faced during the lockdown. The respondents are free to decide about their participation. The proposed study has followed the protocol of COVID-19 for ensuring the protection and safety of interviewer and interviewee. The identities of the respondents will be anonymized by using identifier codes during data collection and write-up. All research participants will be required to participate one-time in a 30-45 minute verbally recorded interviews. The research participants are free to have my contact information for any questions or concerns.

Verbal declaration by the participant: Having carefully heard the details about the research study, in consideration of my appearance/verbal/telephonic interview for the research called “**Impact of Covid-19 on Informal Employment: A case study of women domestic workers in Khyber Pakhtunkhwa, Pakistan”** and other goods and valuable consideration, receipt of which is hereby acknowledged, produced by researchers Dr. Adnan Ahmad Dogar and Dr. Ikram Shah (the **“Researchers”).** I hereby authorize the Researchers to record my name, voice, and participation on audio recorder or mp3 player for use in the above study or parts thereof.

We, (NAMES), warrant and represent that we are over the age of 18 years and that we are free to enter into this Informed Consent**.**

**Furthermore, I also allow the Researchers to use my responses for oral/visual presentations, reports, write-ups, and academic articles.**

**Researchers’ full information**

| Dr. Adnan Ahmad Dogar  Kohsar University Murre  dogaradnan@gmail.com | Dr. Ikram Shah  COMSATS University Abbottabad  ikramshah@cuiatd.edu.pk |
| --- | --- |

**S1 Table Data Availability**

**Detail of Respondents from District Abbottabad**

| **S.N** | **Nature of Work** | **Marital Status** | **Age of Respondent** | **Education Level** | **Household Members** | **Earning Hands** |
| --- | --- | --- | --- | --- | --- | --- |
| 1 | Housemaid | Married | 31-40 Years | Less than Primary | 5 | 2 |
| 2 | Housemaid | Married | 21-30 Years | Primary Level | 8 | 1 |
| 3 | Housemaid | Married | 31-40 Years | Primary Level | 6 | 2 |
| 4 | Housemaid | Married | 21-30 Years | Less than Primary | 6 | 3 |
| 5 | Housemaid | Married | 31-40 Years | No Schooling | 7 | 1 |
| 6 | Housemaid | Married | 31-40 Years | No Schooling | 5 | 2 |
| 7 | Housemaid | Divorced | 51-60 Years | No Schooling | 5 | 1 |
| 8 | Housemaid | Married | 21-30 Years | Less than Primary | 8 | 4 |
| 9 | Laundry-maid | Married | 41-50 Year | Less than Primary | 6 | 2 |
| 10 | Laundry-maid | Married | 41-50 Year | Less than Primary | 5 | 1 |
| 11 | Laundry-maid | Widow | 51-60 Years | No Schooling | 5 | 1 |
| 12 | Laundry-maid | Married | 41-50 Year | Primary Level | 7 | 3 |
| 13 | Laundry-maid | Married | 41-50 Year | Less than Primary | 7 | 3 |
| 14 | Cook | Married | 21-30 Years | No Schooling | 5 | 1 |
| 15 | Cook | Married | 41-50 Year | Less than Primary | 11 | 4 |
| 16 | Cook | Married | 31-40 Years | Less than Primary | 6 | 2 |
| 17 | Cook | Married | 31-40 Years | Less than Primary | 6 | 3 |
| 18 | Child caregiver | Married | 31-40 Years | Secondary School | 7 | 3 |
| 19 | Child caregiver | Single | Up to 20 Years | Less than Primary | 8 | 2 |
| 20 | Child caregiver | Single | 61 and above | Less than Primary | 5 | 1 |

**Detail of Respondents from District Haripur**

| **S.N** | **Nature of Work** | **Marital Status** | **Age of Respondent** | **Education Level** | **Household Members** | **Earning Hands** |
| --- | --- | --- | --- | --- | --- | --- |
| 1 | Housemaid | Married | 21-30 Years | Primary Level | 5 | 1 |
| 2 | Housemaid | Married | 31-40 Years | Less than Primary | 8 | 2 |
| 3 | Housemaid | Married | 31-40 Years | No Schooling | 6 | 1 |
| 4 | Housemaid | Married | 51-60 Years | Less than Primary | 6 | 3 |
| 5 | Laundry-maid | Married | 41-50 Year | Less than Primary | 5 | 1 |
| 6 | Laundry-maid | Widow | 41-50 Year | Primary Level | 5 | 2 |
| 7 | Laundry-maid | Married | 21-30 Years | Primary Level | 7 | 3 |
| 8 | Laundry-maid | Married | 41-50 Year | Less than Primary | 7 | 2 |
| 9 | Laundry-maid | Married | 21-30 Years | No Schooling | 5 | 1 |
| 10 | Laundry-maid | Married | 31-40 Years | Less than Primary | 11 | 4 |
| 11 | Laundry-maid | Married | 41-50 Year | Less than Primary | 6 | 3 |
| 12 | Cook | Married | 31-40 Years | Less than Primary | 6 | 4 |
| 13 | Cook | Married | 41-50 Year | No Schooling | 5 | 3 |
| 14 | Cook | Married | 21-30 Years | Less than Primary | 11 | 1 |

**Detail of Respondents from District Mansihra**

| **S.N** | **Nature of Work** | **Marital Status** | **Age of Respondent** | **Education Level** | **Household Members** | **Earning Hands** |
| --- | --- | --- | --- | --- | --- | --- |
| 1 | Housemaid | Married | 41-50 Year | Less than Primary | 5 | 1 |
| 2 | Housemaid | Widow | 51-60 Years | No Schooling | 7 | 1 |
| 3 | Housemaid | Married | 61 and above | Less than Primary | 6 | 1 |
| 4 | Housemaid | Married | 31-40 Years | Less than Primary | 6 | 2 |
| 5 | Housemaid | Married | 31-40 Years | Less than Primary | 6 | 3 |
| 6 | Housemaid | Married | 31-40 Years | No Schooling | 8 | 2 |
| 7 | Housemaid | Divorced | 51-60 Years | No Schooling | 5 | 1 |
| 8 | Housemaid | Single | Up to 20 Years | Less than Primary | 8 | 2 |
| 9 | Housemaid | Married | 21-30 Years | Less than Primary | 6 | 3 |
| 10 | Laundry-maid | Married | 31-40 Years | Less than Primary | 5 | 2 |
| 11 | Laundry-maid | Married | 21-30 Years | Primary Level | 8 | 1 |
| 12 | Laundry-maid | Married | 41-50 Year | Primary Level | 10 | 3 |
| 13 | Laundry-maid | Married | 41-50 Year | Less than Primary | 7 | 3 |
| 14 | Cook | Married | 21-30 Years | No Schooling | 5 | 1 |
| 15 | Cook | Married | 41-50 Year | Less than Primary | 11 | 4 |
| 16 | Cook | Married | 21-30 Years | Less than Primary | 8 | 4 |
| 17 | Cook | Married | 31-40 Years | No Schooling | 7 | 1 |
| 18 | Cook | Married | 31-40 Years | Secondary School | 7 | 3 |
| 19 | Child caregiver | Married | 41-50 Year | Less than Primary | 6 | 2 |
| 20 | Child caregiver | Married | 31-40 Years | Primary Level | 6 | 2 |
