# Supplementary material for: Impact of Covid-19 on informal employment: A case study of women domestic workers in Khyber Pakhtunkhwa, Pakistan
Source: PLoS One. 2022 Dec 6;17(12):e0278710. doi: 10.1371/journal.pone.0278710 (PMC9725122; doi:10.1371/journal.pone.0278710)
Supplement: Attachment — Submitted filename: Response to Reviewers.docx [file pone.0278710.s002.docx]

**Academic Editor Comments:**

1. **A rebuttal letter that responds to each point raised by the academic editor and reviewer(s). You should upload this letter as a separate file labeled 'Response to Reviewers'.**

A separate file has been uploaded and labeled as “response to reviewers”.

2. **A marked-up copy of your manuscript that highlights changes made to the original version. You should upload this as a separate file labeled 'Revised Manuscript with Track Changes'**

A marked-up copy of manuscript has been uploaded that highlights changes made to the original version of manuscript.

3. **An unmarked version of your revised paper without tracked changes. You should upload this as a separate file labeled 'Manuscript'.**

An unmarked version of revised manuscript has been uploaded without tracked changes.

**Journal Requirements:**

1. **Please ensure that your manuscript meets PLOS ONE's style requirements, including those for file naming. The PLOS ONE style templates.**

The revised version of manuscript has followed the PLOS ONE’s style requirements.

2. **Please provide additional details regarding participant consent. In the ethics statement in the Methods and online submission information, please ensure that you have specified what type you obtained (for instance, written or verbal, and if verbal, how it was documented and witnessed). If your study included minors, state whether you obtained consent from parents or guardians. If the need for consent was waived by the ethics committee, please include this information.**

In methodology section on line 175-179 detail has been added to address this comment by explaining the way a verbal consent has been taken from the study participants. In this study, minors were not included in data collection process.

3. **In your Data Availability statement, you have not specified where the minimal data set underlying the results described in your manuscript can be found. PLOS defines a study's minimal data set as the underlying data used to reach the conclusions drawn in the manuscript and any additional data required to replicate the reported study findings in their entirety. All PLOS journals require that the minimal data set be made fully available.**

The Data Availability statement has been updated as per the PLOS ONE requirements.

4. **Please include your full ethics statement in the ‘Methods’ section of your manuscript file. In your statement, please include the full name of the IRB or ethics committee who approved or waived your study, as well as whether or not you obtained informed written or verbal consent. If consent was waived for your study, please include this information in your statement as well.**

The full ethics statement has been added in the Material and Methods section on line 163-167.

5. **We note that Figure 1 in your submission contain map images which may be copyrighted. All PLOS content is published under the Creative Commons Attribution License (CC BY 4.0), which means that the manuscript, images, and Supporting Information files will be freely available online, and any third party is permitted to access, download, copy, distribute, and use these materials in any way, even commercially, with proper attribution.**

Figure 1 has been removed from manuscript.

6. **We note you have included a table to which you do not refer in the text of your manuscript. Please ensure that you refer to Table 3 in your text; if accepted, production will need this reference to link the reader to the Table.**

The Table 3 has been referenced in the text on line 206.

7. **Please include captions for your Supporting Information files at the end of your manuscript, and update any in-text citations to match accordingly. Please see our Supporting Information guidelines for more information.**

Caption has been added to Supporting Information file as “S1 Appendix”.

**Additional Comments:**

1. **Please abide by the journal guideline.**

Manuscript has been revised as per the journal guidelines.

2. **What are statistical methodologies?**

In methodology section on line 188-189 the details of statistical analysis have been given.

3. **Check the grammar and English language editing.**

The revised version of manuscript has been proofread and edited by English language expert.

**Reviewer # 1:**

1. **Regarding 3, not applicable, the data is qualitative interview with human beings and therefore should not be made fully available to anyone to preserve anonymity.
   The study is original, timely and relevant, and presents important findings on an under-studies sector. The qualitative approach is also relevant and adds to the understanding of perceptions and needs of domestic workers in Pakistan. Finally, this gives a strong basis for policy recommendations.**

We appreciate the time and efforts of esteemed reviewer for the valuable insight and feedback.

**Reviewer # 2:**

1. **The level of this manuscript is lower than PLOS ONE stander. However, my recommendation to the authors to increase the number of participants in future studies.**

In future studies the number of respondents will be increased as statistically appropriate.

2. **What is the statistical software used for this manuscript? (Not clear).**

The details of statistical software have been added on line 189-190.

3. **Authors should carefully follow instructions for manuscript preparation and ensure that the manuscript is proofread before submission (follow Submission Guidelines for PLOS ONE).**

During the revised manuscript preparation, due consideration has been paid to ensure PLOS ONE submission guidelines and proofread aspect.

4. **This manuscript needs English editing (you should include certificate).**

The revised manuscript has been proofread and edited by English language expert. All the changes have been indicated in “marked-up copy of manuscript” file.
